# Identification of Novel Modalities Through Bibliometric Analysis for Timely Development of Regulatory Guidance: A Case Study of T Cell Immunity

**DOI:** 10.3389/fmed.2021.756870

**Published:** 2021-10-11

**Authors:** Ai Fukaya-Shiba, Kouhei Otsuka, Hajime Sasaki, Mayumi Shikano, Rika Wakao

**Affiliations:** ^1^Center for Regulatory Science, Pharmaceuticals and Medical Devices Agency, Tokyo, Japan; ^2^Faculty of Pharmaceutical Sciences, Tokyo University of Science, Tokyo, Japan; ^3^Institute for Future Initiatives, The University of Tokyo, Tokyo, Japan

**Keywords:** regulatory science, horizon scanning, drug development, citation network, regulatory guidance, immunotherapy, T cell therapy, novel modality

## Abstract

**Background:** The mission of medicines regulatory agencies is to ensure the timely access of innovative products for patients to improve public health. Thus, regulators should foresee evolving technologies and build expertise prior to reviewing innovative products. Novel modalities and new classes of therapeutics in biological or cell-based products represent a regulatory challenge because of knowledge gaps, as exemplified by the unexpected cytokine release syndrome in the first-in-human clinical trial of the CD28 super-agonist. Meanwhile, recent treatments harnessing T cell co-signaling pathways provide an opportunity for investigation. Therefore, this study aimed to systematically identify and evaluate novel modalities for T cell immunity to assess the need for regulatory guidance.

**Methods:** A PubMed search was carried out using the query, “immun^*^ AND t lymph^*^” to select publications. Subsequently, a citation network was created, followed by clustering and text mining to identify the modalities and classes of therapeutics under development.

**Results and Discussion:** Analysis of the top 20 clusters revealed research domains characterized by keywords such as immune checkpoint antibody, chimeric antigen receptor (CAR)-T cells, microbiota, exosome, regulatory T cells, unconventional T cells, and vaccines. After reviewing the pharmacological concepts, clinical trial information, and available guidance, we presented a perspective on the future development of guidance for these domains.

**Conclusion:** Bibliometric analyses identified a set of innovative modalities targeted for drug development with which regulatory guidance is going to catch up. This strategy could help in the successful development of upcoming modalities to ensure readiness for clinical application as part of horizon scanning.

## Introduction

Our mission as medicines regulatory agency is to protect and promote public health. We achieve our mission through regulatory science, which underlies the objective evaluation of the safety, efficacy, and quality of medical products and supports science-based decision-making. The development of standards and regulatory guidance accelerates product development and regulatory review to make innovative products available to the public in a timely manner. At the same time, regulatory agencies are confronted with emerging technologies that may have issues beyond the expertise gained from existing medical products. Novel modalities in biological or cell-based products represent a regulatory challenge in terms of efficacy, safety, and quality because of the heterogeneous nature of the product and the multifaceted mode of action. For example, the use of intestinal microbiota as biological products poses a gap to be filled, as they do not reach the systemic circulation but rather modulate mucosal immunity (1). To respond to innovation, medicines regulators worldwide, including in Europe (2) and Japan (3), explore many ways for horizon scanning and cooperate via an international framework, the International Coalition of Medicines Regulatory Authorities (ICMRA) (4). As described previously, it is common to use scientific literature, committees, expert groups, the web, and Delphi methodologies to identify innovation (5). Thus, a comprehensive and transparent methodology is required.

To complement foresight capacity, text mining of a dataset of scientific publications provides a tool for the early identification of emerging technologies, as discussed for Tools for Innovation Monitoring in Europe which makes an overall science survey (6). Text mining technique has extensively been used by policy-makers (6, 7). A combination of text mining and network analysis reported the emergence and evolution of research fronts in biomedical areas (8–11). This strategy, using bibliometric analysis, has the advantage of being supported by scientometric evidence and elucidates paradigms or key elements organizing innovation.

A caveat for searching a database is to determine the appropriate “search term,” which captures the panoramic view of how the key elements of the target field are organized. One solution is to select an encompassing search term that captures the co-evolution of related paradigms (10).

Herein, we focused on the pharmacologic interventions that have been developed for T cell immunity as a case study of bibliometric analysis for horizon scanning. T cells play pivotal roles in the immune system and have therapeutic potential against cancer, autoimmune and/or infectious diseases, and inflammatory conditions. The ability of T cells to form “memory” cells is the fundamental basis of vaccination; however, they are also responsible for harmful reactions such as graft-versus-host disease (GvHD) or donor cell rejection in allogeneic transplants. The first-in-human clinical trial of TGN1412 (monoclonal antibody to co-stimulator CD28), which caused serious adverse effects, highlighted the critical need for regulating the therapeutic ability of T cells and their destructive potential (12). Recently, however, harnessing T cell co-signaling pathways to re-ignite T cell immunity has achieved the practical use as two modalities: one is cellular modality targeting cancer antigens through highly activated chimeric antigen receptor (CAR)-T cells, the other is antibody re-activating endogenous quiescent T cells through checkpoint blockade. These new treatment paradigms prompted us to investigate the development of the core modality in T cell immunity.

To systematically identify novel modalities, we took three steps; network formation with direct citation links, followed by dividing the network into several clusters, finally extracting the characteristic keywords of each cluster. These steps allow us to grasp the overall landscape of T cell immunity, position and interpret each cluster as a distinct technical domain and analyze the targeted clusters with keywords.

This study aimed to explore the possibility of a citation network and clustering in identifying modalities and classes of therapeutics under development. We hypothesized that bibliometric analyses would reveal clusters of distinct modalities in T cell immunity, which would warrant regulatory guidance.

## Methods

### Citation Network Analysis and Text Mining

The search query “immun^*^ AND t lymph^*^” was selected for PubMed search, which yielded seven key articles (13–19) in the research history of immune checkpoint inhibitors based on the official page for the Nobel Prize in Physiology or Medicine 2018 awarded to Dr. James P. Allison and Dr. Tasuku Honjo (20).

We retrieved 134,361 publications from PubMed (published up to December 2020), of which 92,731 (69.0 %) resulted in a citation network by extracting the largest connected component from all linkage components via direct citation of publications. The start date was not specified to collect publications in the PubMed as much as possible. The year of the oldest paper in the largest connected component was 1970.

As shown in Supplementary Figure 1, after forming a citation network from PubMed publications, it was converted into an unweighted network with publications as nodes and citation relationships as links. The network was then divided into several clusters using the topological clustering method with modularity maximization (Louvain method) (21–23). Subsequently we computed the term frequency-inverse cluster frequency (TFICF) to extract the characteristic keywords of each cluster. TF provides a measure of the importance of a term in a particular sentence. ICF provides a measure of the general importance of a term. The TFICF of a given term i in a given cluster j is calculated as follows:


$$\begin{array}{l} TFICF=tf_{i,j}\cdot icf_{i}=tf_{i,j}\cdot \log\left(N/cf_{i}\right) \end{array}$$

where N is the total number of sentences. TFICF reflects how important and specific a word is to a cluster in comparison with the collection of clusters. The TFICF value increases proportionally to the number of times a word appears in the targeted cluster and is offset by the number of clusters that contain the word. TFICF differentiates the characteristic words in a cluster from words that appear in general. The keywords, ranked in the top 20 TFICFs related to harnessing T cells for therapeutics, were listed.

### Other Information

Clinical trial information was collected from ClinicalTrials.gov; regulatory guidance information, as of July 2021, was retrieved from the FDA, EMA, and PMDA websites.

## Results

We analyzed a citation network of publications obtained from PubMed, and 38 clusters were formed. The clusters were arranged in descending order of the number of included constituent papers (Supplementary Figure 1); the top 20 clusters were used for subsequent analyses, which covered 95.3% of papers in the citation network.

Table 1 summarizes the cluster keywords, ranked in the top 20 TFICFs, on the recently developed modalities and research fields. Clusters 1, 2, 3, 11, 13, 16, and 20 were chosen because they contained keywords related to the use of T cells for therapeutics. Cluster 1 contained keywords related to immunotherapy, including vaccines, immune checkpoints, CARs, and cytotoxic T lymphocytes. This cluster also had “oncolytic” as a keyword with a lower TFICF. Cluster 2 consisted of keywords related to mucosal immunity, such as microbiota, intestinal, and dendritic cells (DCs). In addition, clusters 1 and 2 were sub-clustered due to the large volume of publications to extract specific topics, showing the detailed character of each cluster. Cluster 3 included keywords on regulatory T cells (Tregs), autoimmunity, and tolerance, while cluster 11 showed exosomes at the top of TFICF. The keywords for cluster 13 were characterized by unconventional T cells, such as invariant NKT (iNKT) cells and mucosal-associated invariant T (MAIT) cells. The keywords for cluster 16 included coronavirus, vaccine, and severe acute respiratory syndrome, while those for cluster 20 comprised mesenchymal stem cells (or mesenchymal stromal cells) (MSCs). Our analysis identified novel modalities classified into each cluster in the citation network.

**Table 1:** Characterization of the identified clusters by TFICF keywords.

**Relevant cluster or sub-cluster**	**Average year**	**Number of papers**	**Top keywords on the modalities and key elements in research fields**	
				**Diseases and etiology**
Cluster 1	2011	14,538	immunotherapy, vaccine, immune checkpoint, ipilimumab, CAR, CD8, CTL	tumor, melanoma
1-1	2011	2,286	programmed death (PD), CTLA, TIM, TIGIT, VISTA, ICOS, CD28, CD155	tumor
1-3	2017	1,606	immune checkpoint inhibitor, TMB, neoantigen, microenvironment, STING	tumor, melanoma, BRAF, MCPyV
1-6	2014	1,231	anti CTLA, immune checkpoint inhibitor, ipilimumab, nivolumab, tremelimumab, IRAEs	tumor, melanoma, hypophysitis
1-8	2011	1,084	CAR, bispecific antibody, adoptive	tumor, leukemia, WT1
Cluster 2	2008	11,087	peptide, microbiota, intestinal, mucosal, pylorus, dendritic cell (DC), tolerance, IFN, gamma, CD4	autoimmune, infection, allergen, inflammation
2-3	2012	1,376	intestinal microbiota, probiotic, colitis, mucosal, Treg	IBD, hepaticus, ILFs
Cluster 3	2011	9,628	Treg, foxp3, tolerance, CD4, CD25	tumor, autoimmune, T1D
Cluster 11	2008	2,471	exosomes, TCR	tumor
Cluster 13	2007	1,977	iNKT, NKT, MAIT, alpha GalCer, CD1d	tumor
Cluster 16	2010	1,255	vaccine	covid, coronavirus, severe acute respiratory syndrome (SARS), sars cov infection, tumor
Cluster 20	2011	824	MSC, ASCS	ITP

*The following keywords were obtained as abbreviations. CAR, chimeric antigen receptor; CTL, cytotoxic T lymphocyteTIM, T cell immunoglobulin and mucin domain; PD, programmed death; CTLA, cytotoxic T lymphocyte associated antigen; TIM, T cell immunoglobulin and mucin domain; TIGIT, T cell immunoreceptor with immunoglobulin and ITIM domains;, VISTA, V-domain immunoglobulin suppressor of T cell activation; ICOS, inducible T cell costimulatory; TMB, tumor mutation burden; STING, stimulator of interferon genes; MCPyV, Merkel cell polyomavirus; IRAEs, immune-related adverse events; IFN, interferon; Treg, regulatory T cell; IBD, inflammatory bowel disease; ILFs, isolated lymphoid follicles; T1D, type 1 diabetes; TCR, T cell receptor; iNKT, invariant natural killer T (cell); MAIT, mucosal associated invariant T (cell); GalCer, galactosylceramide; MSC, mesenchymal stem cell; ASCS, adipose derived stem cells; ITP, idiopathic thrombocytopenic purpura*.

We characterized the research trends in each cluster by selecting papers on drug development or translational research, which have been published recently (mainly in the last 5 years). The clusters, categorized by modality, are summarized in Table 2. In addition, the clinical study information for each modality was supplemented to validate drug development.

**Table 2:** Research trends in each cluster.

**Modality**	**Relevant cluster or sub-cluster**	**Research trend and technological class**	**References in each cluster**	**Clinical development (excluding the approved products)**
immune check point antibody	Cluster 1 1–1	Immune check point modulators Inhibitory immune checkpoints PD-1, CTLA-4, LAG-3, TIM-3, TIGIT, VISTA	(24–27)	(28) LAG-3, TIM-3, (29) TIGIT; Phase III as of July 2021, (30) VISTA
		Stimulatory immune checkpoints CD28, OX40, 4-1BB, GITR, CD40, ICOS		(31) OX40, 4-1BB, (32) GITR, (33) CD40
	1–3	Response and resistance to immune check point therapy tumor microenvironment (TME), TMB, neoantigen	(34–38)	(39) exploratory TMB, (40) IFN-γ-related gene expression signatures, (41) IFN-γ production within the TME, (42) microbiota, (43) fecal microbiota transplantation
	1–6	IRAEs and immunotherapy combination cancer vaccines, oncolytic viruses, adoptive cell therapy and checkpoint blockade	(44–47)	(48) melanoma antigens, (49) autophagosome vaccine, (50) cancer vaccine, (51) oncolytic virus
CAR-T cells	1–8	Engineered T cells and Bispecific T cell engager CAR, bispecific antibody, TCR-engineered T (TCR-T) cells	(52–55)	(56, 57) TCR-T cells; solid tumor, (58) prime CAR-T cells; solid tumor
microbiota	Cluster 2 2–3	Manipulation of gut microbiota for the treatment of diseases microbiota, commensal bacteria, intestinal microbiota, IBD	(59–62)	(63) *Clostridioides difficile* infection, (64) Crohn's disease, (65) melanoma, (66) food allergy
T cell subtype	Cluster 3	Treg for immune-suppression Treg, FOXP3, CD25	(67–69)	(69) T1D, (70) minimizing immune suppression in kidney transplantation
	Cluster 13	Unconventional T cell for immunomodulation iNKT cell, MAIT cell, cd1d, alpha GalCer	(71–74)	(75) iNKT cells, (76) allogeneic iNKT cells
vaccine	Cluster 16	SARS-CoV-2 and T cell response COVID-19, coronavirus, vaccine, SARS	(77–79)	–
exosome	Cluster 1 Cluster 11 Cluster 20	Immunoregulation by exosomes	(80, 81) (82, 83) (84, 85)	(86) DC-derived, (87) MSC-derived

*LAG-3, lymphocyte activation gene 3; GITR, glucocorticoid-induced tumor necrosis factor-related protein; the other abbreviations are listed in Table 1*.

Recent studies on immune checkpoint antibodies were classified mainly into sub-clusters 1-1, 1-3, and 1-6.

- Sub-cluster 1-1 included papers on a similar class of immune checkpoint modulators, i.e., inhibitory or stimulatory immune checkpoints. Although antibodies against the co-inhibitory receptors, cytotoxic T lymphocyte antigen 4 (CTLA-4) and programmed cell death 1 (PD-1), exhibit prominent efficacy in several cancer indications, only 20% of cancer patients respond to single-agent checkpoint inhibitors (24). Accordingly, an increasing number of studies in developing novel checkpoint modulators that can reverse the blockade or rejuvenate T cell immunity and their combination has been observed (24–27). Various immune checkpoint modulators, such as lymphocyte activation gene 3 (LAG-3), TIM-3, TIGIT, VISTA, OX40, 4-1BB, GITR, and CD40, have been reported in clinical trials, in combination or compared with anti-PD-1 or anti-CTLA-4 therapy (28–33). Given that cancer and chronic infections share common features, such as chronic exposure to antigens and the development of exhausted effector T cells, there is growing interest in strategies that apply immune checkpoint inhibitors to chronic viral infections (25, 26). In both cases, the therapeutic goal is to rejuvenate T cell immunity to eradicate tumors or virus-infected cells. On the other hand, in transplantation settings, the focus on manipulating T cell co-signaling is to induce tolerance rather than rejuvenation (27).
- Sub-cluster 1-3 contained issues of response and resistance to immune checkpoint blockade, tumor microenvironment (TME), and tumor mutation burden, which have been proposed as predictive biomarkers for the response to immune checkpoint blockade (34, 39). Loss of the interferon (IFN)-γ pathway has been reported as a mechanism responsible for the lack of clinical responses to checkpoint blockade in some patients (35, 40). A phase II clinical trial is underway to investigate the combination of checkpoint blockade and IFN-γ production within the TME (41). Cancer vaccines require co-treatments to overcome immune evasion and immune-suppressive microenvironments (36). Another study pointed out that a personal, multi-peptide, neoantigen vaccine for melanoma was effective alone or in combination with checkpoint blockade (37). This cluster also included a report on boosting checkpoint blockade with microbiota therapy in preclinical models (38) and clinical studies (42, 43).
- Sub-cluster 1-6 contained issues regarding immune-related adverse events, specifically those related to immune checkpoint blockade (44, 45) as well as a combination of cancer immunotherapy, including cancer vaccines, adoptive cellular immunotherapy, and oncolytic viruses, to improve clinical response and minimize toxicities (46, 47). Clinical studies on combination therapy of cancer vaccines (48–50) or oncolytic viruses (51) have also been reported.

The papers on engineered T cells and bispecific antibodies were predominantly compiled in sub-cluster 1-8. T cells genetically engineered to express artificial receptors, such as CARs, have been the subject of intense scrutiny (52, 53). The mechanism of bispecific antibodies is similar to that of CARs: it involves bridging two target cells, thereby bringing immune effector cells into close contact with particular tumor-associated antigens to facilitate cell killing (54). Compared to CAR-T cells in B-cell malignancies, the treatment of solid tumors with CAR-T cells is less effective. CAR-T cell treatment targeting EGFRvIII in glioblastoma resulted in antigen escape because of selection pressure favoring expansion of a subset of tumor cells that lacked the targeted antigen in the clinical trial (55). NY-ESO-1-specific T cell receptor-engineered T (TCR-T) cells have generated clinical responses in patients with synovial cell sarcoma and have received Sakigake and Orphan regenerative medical product designation in Japan (56, 88, 89). Clinical studies on the treatment of solid tumors with TCR-T cells targeting MAGE-A4 (57) or CAR-T cells targeting glypican 3 (GPC3) have been reported (58). Cluster 1 showed research trends on enhancing the antitumor activity of immunotherapy and expanding disease targets, while minimizing adverse events based on the molecular mechanism of immune checkpoint blockade and engineered T cells.

We focused on sub-cluster 2-3 in cluster 2, since it contained unique papers on mucosal immunity, including studies involving intestinal microbiota and commensal bacteria. Although this sub-cluster did not have many recent publications, it included those relevant to the clinical development of microbiota-based products. The top-cited papers describe how commensal microbiota affect specific host T cells (59, 60). A subsequent study reported a preclinical study on the isolation of Treg-inducing bacterial strains from human microbiota (61). Together with the study by Sivan et al. (38), studies on the mechanism of microbiota-host interaction provided evidence regarding the therapeutic potential of selected microorganisms for inflammatory disease and cancer immunotherapy (62). Clinical studies designed to assess the efficacy of microbiota in addressing specific diseases have also been reported (63–66).

Cluster 3 involved studies on Tregs. Sharabi et al. summarized clinical trials of therapies administering Tregs to treat autoimmune diseases, transplantation, and cancer (67). Practical issues related to the isolation and manufacture of Tregs for cell therapy have been noted (68). Clinical studies, on the use of Tregs in treating type 1 diabetes (69) and kidney transplantation (70), have been reported. This cluster revealed clinical applications and hurdles for Treg-based cell therapy.

Cluster 13 comprised papers on iNKT cells that recognize specific glycolipid antigens (alpha galactosylceramides) presented by CD1d protein (71). Innate-like or unconventional T cells include iNKT, MAIT, and γδ T cells, which recognize lipids, vitamin B2 metabolites, and specially modified peptides, respectively. The properties of these cells encompass innate and adaptive immune responses against cancer and infectious diseases (72, 73). Notably, unconventional T cells are considered as non-traditional adjuvants to improve vaccine efficacy and are capable of stimulating a wide array of immune cells (74). Phase I clinical studies on iNKT cells have also been reported (75, 76).

Cluster 16 was distinct in that it consisted of papers on coronavirus and vaccines. The number of papers in this cluster reached a maximum in 2020 (Supplementary Figure 1). Severe acute respiratory syndrome coronavirus 2 (SARS-CoV-2)-specific T cells in patients with acute respiratory distress syndrome have been characterized (77). The kinetics of immune responses, in relation to the clinical and virological features of a patient with mild-to-moderate coronavirus disease 2019 (COVID-19), have been reported (78). Kim et al. discussed recent evidence on the adaptive immune response against SARS-CoV-2 and its potential implications for the generation of memory responses from the vaccine viewpoint (79).

Clusters 1, 11, and 20 contained papers on extracellular vesicles (EVs), including exosomes which have been the subject of intense scrutiny, with respect to therapeutic applications, because of their capacity for intercellular communication in modulating immune responses (82). Plasma-derived exosomes were found to be predictive of non-invasive biomarkers of immune dysfunction in head and neck cancer (83). Exosomes secreted by DCs have been sought as therapeutic antitumor vaccines in clinical studies (80, 86), while engineered tumor cell-derived exosomes potentiated DC immunogenicity and long-lasting antitumor immunity in preclinical models (81).

Cluster 20 contained papers on MSCs. Stem/progenitor cell-derived EVs exert immuno-regulatory effects on immune cells, such as natural killer (NK) cells, DCs, and T cells (84). The immuno-modulatory activity of MSC-derived exosomes was compared with that of parental MSCs (85). Respiratory diseases were the most common indication in clinical trials registered for MSC-derived EVs therapeutics (90). Clinical studies of exosomes carrying siRNA (87) have also been reported.

Table 3 lists the regulatory guidance documents issued for each modality identified in the present study, as well as the time of approval of the first product. Guidance documents were available for cancer vaccines, oncolytic viruses, microbiota, CAR-T cells and bispecific antibodies, and unavailable for immune checkpoint inhibitors and exosomes (as of July 2021).

**Table 3:** Guidance issued for cutting-edge modalities.

**Modality**	**Guidance (published year)**	**Reference**	**Product approved year**
cancer vaccine	FDA (2011)	(91)	US in 2010
oncolytic virus	ICH (2009)	(92)	US in 2015
microbiota	FDA (2016), PMDA (2021, planning)	(93)	(^*^)
CAR-T cells	EMA (2020)	(94)	US in 2017
bispecific antibody	FDA (2021)	(95)	US in 2014
exosome	PMDA (2022, planning)	–	(^*^)

* *Guidance needs to be developed or updated*.

## Discussion

Our investigation revealed citation network and clustering captured the structure of T cell immunity field as distinct clusters. Subsequently, our review of knowledge in each cluster brought understanding of research fronts of major modalities. These steps allowed us to assess the needs to develop regulatory guidance for each modality. Our method provides an effective tool for regulators to identify state-of-the-art research fronts to develop guidance documents in a timely manner, minimizing the gap between scientific innovation and product review.

### Bibliometric Snapshot of the Evolving Paradigm

Using the “immun^*^ AND t lymph^*^”query, we identified several clusters that contained coherent groups of immunological paradigms. The construction of a network of direct citations between papers is useful for structurally grasping the origin of knowledge in the field, and the network clustering method can be used to extract distinct sub-regions. It is reasonable that cluster 1, the largest cluster, consisted of the immune checkpoint blockade and CAR-T cells sub-clusters, and provided an abundance of data for immunotherapies and interconnected concepts. The addition of a co-stimulatory domain into the second-generation CAR greatly enhanced efficacy over that of the first-generation CAR (55), leading to FDA approval. We also observed some intra- and inter-cluster-linked papers contributing to the conceptual framework. The top-cited paper in cluster 1 described a phase III trial demonstrating survival benefit in patients undergoing anti-CTLA4 therapy (96) which has been cited by a preclinical study in sub-cluster 1-3 that revealed the mechanism of tumor-specific mutant antigen, and the target of checkpoint blockade therapy, thus proposing personalized cancer-specific vaccines (97). While this preclinical study is cited by a review on combination therapy (98) in sub-cluster 1-1, it is also cited by a review on CAR-T cells for solid tumors (55) in sub-cluster 1-8. Therefore, our method allows us to trace how a paradigm is developed.

### Assessing the Need of Regulatory Guidance

Regulatory agencies must build their expertise prior to reviewing forthcoming products developed from evolving technologies, to ensure availability of innovative products to patients in a timely manner. We collected guidance documents that show current regulatory thinking on chemistry, manufacturing, and controls (CMC) as well as preclinical and clinical issues for specific modalities.

Developing guidance for EVs is the top priority among the identified modalities, since there is no guidance available. The PMDA Science Board, a high-level consultative body that discuss the scientific aspects of medical product review, will develop points to consider (PTC) for EV-based products in a year (90). Although EVs, including exosomes, have drawn attention as potential therapeutics, their quality requirements are yet to be addressed by regulatory bodies. Given the high congruence of size and behavior between EVs and viruses, any virus present in the materials or manufacturing process could be enriched in the final product. Thus, a sound basis for assessing EV-based products must be established.

As a high priority, the updated guidance of microbiota as biotherapeutic products is needed, as there is no product approved. The guidance for live biotherapeutic products was developed by the FDA in 2012 and subsequently revised in 2016, while in Europe, in the absence of EU guidelines, a roadmap for safety assessment was proposed (1). The PMDA Science Board will be reporting PTC on live biotherapeutic products based on the latest knowledge. There is a need to continuously update the regulatory guidance based on scientific advances made in the field, and such documentation can facilitate the development of novel modality-based products.

As for CAR-T cells, more specialized guidance could be considered. While EMA provided clinical considerations on CAR-T cells in hemato-oncology in 2020 (94), it is reasonable to expect multifaceted issues relevant to CAR-T cells will be addressed, including their use in the treatment of solid tumors (55) or allogeneic genome-edited CAR-T cells (99). Allogeneic CAR-T cells, using T cells from healthy donors, would provide timely access to the treatment for patients, with stable quality, avoiding the problem of T cell exhaustion inherent to cancer patients. Genome-editing of endogenous TCR is undertaken to overcome the harmful effects inherent to these molecules, such as GvHD (donor cells attacking recipient tissue). However, genome-editing is accompanied with safety concerns regarding off-target effects, as described in the PTC of the PMDA Science Board (100). Besides the structure-engineering of CAR, consideration as alternative sources of T cells, such as NK cells, unconventional T cells, or Tregs should also be regarded, as discussed below.

Because of the HLA-independent monomorphic nature of CD1d or MHC class I-related protein (MR1), which constrains iNKT or MAIT cell development, unconventional T cells can be potential CAR carriers. These cells may provide a platform for CAR-T cell therapy in allogeneic settings that do not induce GvHD (101). In addition, these cells may serve as antitumor effector cells since they represent an effector and memory phenotype.

We should carefully monitor the evolution of the translational potential of these cells to assess the need for regulatory guidance.

Regarding other identified modalities, the priority to develop guidance is not high, given that the guidance documents are available, and the products were approved. FDA guidance for cancer vaccines and ICH consideration for oncolytic viruses were issued close to the product approval time, thereby ensuring timely patient access. FDA guidance for bispecific antibodies was issued after product approval, implying the intention to inform the development of other types of bispecific or multi-specific protein products.

Despite the tremendous impact on clinical use, there has been no specific guidance for immune checkpoint inhibitor development. We assume that this is because the regulatory pathway for evaluating monoclonal antibodies is well-established. Instead, the management of immune-related adverse events, which are distinct from those of conventional cytotoxic and molecular-targeted drugs, has drawn attention, as discussed (44, 45). Recent progress in the development of immunotherapy has altered the strategy for developing anti-cancer drugs, necessitating revision of the guideline for these clinical evaluations in Japan (102).

### From a Different Perspective

From the regulatory perspective of T cell immunity, it is imperative to discuss the consequence of the TGN1412 clinical trial. TGN1412, a super-agonistic monoclonal antibody specific for CD28 (CD28SA) that is intended to activate Treg cells, was found to be therapeutically active in multiple rodent models of autoimmunity. However, a phase I trial of TGN1412 failed to induce Tregs but instead caused life-threatening cytokine storms in healthy volunteers (12, 103). In response to these results, regulators committed to minimizing the risk of serious adverse reactions by publishing guideline and its update (104). It had repercussions not only on mitigating risks for first-in-human trials, but also on improving the translational potential of laboratory animals. A recent study showed that laboratory mice failed to mimic the phenotype of human subjects, whereas wildlings with natural microbiota closely mirrored human immune responses (105), indicating the importance of antigenic experience in immune cells when considering translational research. Immune phenotypes and functions emerge from the combination of genetics, epigenetics and environment, including microbiota (106). These findings might trigger a revisit of the ICH S6 guideline (107) on preclinical safety evaluation of biotechnology-derived pharmaceuticals.

Apart from detecting novel modalities, our citation network compiled scarce papers on TGN1412, sporadically found in clusters 1, 5, and 6 with the keyword, “TGN1412,” in TFICF (108–113). Although one review described TGN1412 in the perspective of T cell manipulation technology in 2012 (113) and another study reported a humanized mouse model (109), we manually filled the gap in scientific progress in the subsequent years. We admit that technological concepts with high volumes of linked papers are easy to detect, while concepts with limited research resulting in papers with low linkage need careful consideration.

### Limitation

We acknowledge that our analysis of the network structure does not have predictive power for future innovation. Other information, such as patents and budgets of the target modalities, should be considered to create a cohesive plan for timely roadmaps. Another limitation of our study largely reflects the nature of the clustering. Extracting publications by the largest connected component from all linkage components might result in possible missed insights. This strategy may exclude relevant papers with weak linkages, which could be related to the intended objectives. For example, groundbreaking research on the translatability of wildling mice with natural microbiota (105) was not included in the clusters analyzed. Likewise, most recent papers could not be recovered in the citation network because of the low frequency of citations, as observed for TGN1412-related papers. Such possibilities need to be carefully considered. Thus, it is important that bibliometric results be seen as starting points for subsequent exploratory analyses and reviews.

## Conclusion

The present bibliometric analysis captured a set of innovative modalities targeted for drug development and revealed several classes of therapeutics of importance. The keywords in the clusters highlight the roadmap for the timely development of regulatory guidance as well as features of research trends that provide important perspectives for subsequent consideration. The citation network offered an efficient and transparent exploratory analysis for horizon scanning that could be considered a starting point for further review and evaluation.

## Data Availability Statement

The raw data supporting the conclusions of this article will be made available by the authors, without undue reservation.

## Author Contributions

RW contributed to conception, design of the study, and wrote the manuscript. HS organized the database. AF-S and KO collected data. AF-S performed data analysis. AF-S and MS reviewed and edited the manuscript. All authors contributed to the article and approved the submitted version.

## Conflict of Interest

The authors declare that the research was conducted in the absence of any commercial or financial relationships that could be construed as a potential conflict of interest.

## Publisher's Note

All claims expressed in this article are solely those of the authors and do not necessarily represent those of their affiliated organizations, or those of the publisher, the editors and the reviewers. Any product that may be evaluated in this article, or claim that may be made by its manufacturer, is not guaranteed or endorsed by the publisher.
