# Supplementary material for: Identification of Novel Modalities Through Bibliometric Analysis for Timely Development of Regulatory Guidance: A Case Study of T Cell Immunity
Source: Front Med (Lausanne). 2021 Oct 11;8:756870. doi: 10.3389/fmed.2021.756870 (PMC8544749; doi:10.3389/fmed.2021.756870)
Supplement: Supplementary file 1 [file Data_Sheet_1.pdf]

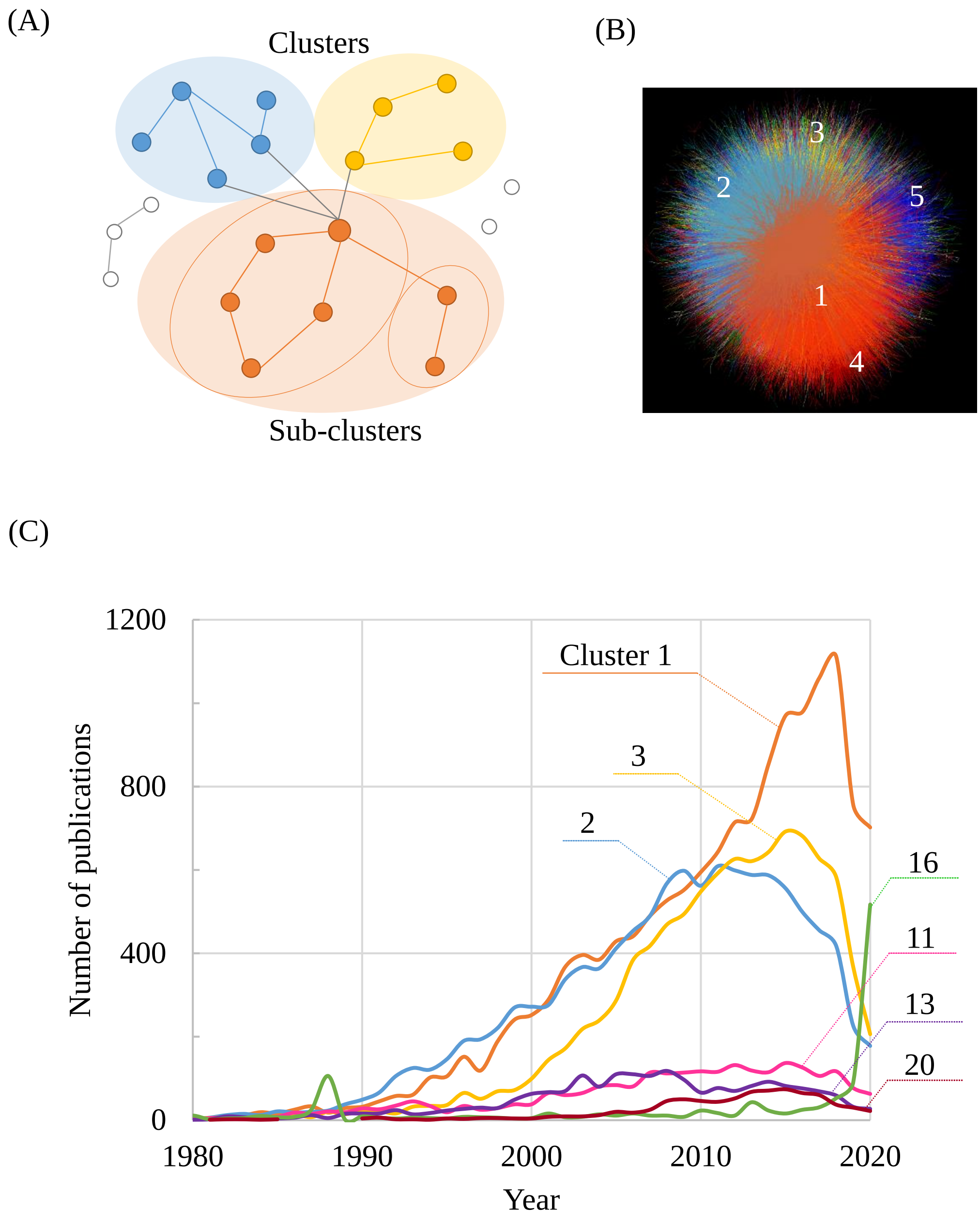

Supplementary Figure 1. Clustering and visualization of the academic landscape of T cell immunity research publications.

The direct citation network of the research publications was created using the target dataset obtained from the PubMed database. Subsequently, the network in the largest connected component was divided into several clusters using the topological clustering method with modularity maximization; large clusters were further divided into sub-clusters (A). The clusters were numbered in descending order of the number of included constituent papers, and the academic landscape was visualized (B). The number of yearly publications for the seven clusters containing targeted modalities is also displayed (C).
